# Predicting Recombinant mRNA Loading into Extracellular Vesicles: Insights from CD81 Fusion Constructs

**DOI:** 10.3390/ijms27083484

**Published:** 2026-04-13

**Authors:** Alessia Gabardi, Elena Gurrieri, Giulia Carradori, Dalia Tarantino, Vito Giuseppe D’Agostino

**Affiliations:** Department of Cellular, Computational and Integrative Biology (CIBIO), University of Trento, Via Sommarive 9, 38123 Trento, Italy; alessia.gabardi@unitn.it (A.G.); elena.gurrieri@unitn.it (E.G.); giuliacarra99@gmail.com (G.C.); dalia.tarantino@unitn.it (D.T.)

**Keywords:** tetraspanin, recombinant RNAs and proteins, vesicle sorting

## Abstract

Extracellular vesicles (EVs) are increasingly explored as vehicles for intercellular communication and the delivery of functional molecules, such as RNA. Recent studies have identified transcript-intrinsic features that influence EV-RNA sorting, including sequence length/complexity and coding probability. However, predicting the enrichment of coding transcripts into EVs remains exploratory. Using the workflow we previously described for the characterization of CD81 fusion constructs, we measured the vesicular distribution of recombinant transcripts transiently expressed in HEK293T cells, yielding protein cargo that mirrored the intracellular abundance. We included the CD81Δ and E7 constructs to obtain insights into the potential correlation between transcript length and EV distribution. We observed that EV-RNA levels do not scale proportionally with intracellular abundance, unlike the corresponding protein cargo. We consider cumulative RNA structure, sub-cellular dynamics, post-transcriptional modifications, and RNA-binding protein interactions as necessary factors that may dictate mRNA recruitment into EVs independent of transcript length, possibly inspiring new trajectories to maximize EV-RNA loading strategies.

## 1. Introduction

Extracellular vesicles (EVs) are secreted membranous particles comprising small and large vesicle populations [1]. EVs are recognized as mediators of intercellular communication and may play roles in physiological and pathophysiological processes, including inflammation, immune regulation, cancer, and neurodegenerative diseases [2,3,4]. EVs can be isolated from biological fluids and represent a promising source of liquid biopsy, enabling multi-analyte diagnostic approaches [5]. In parallel, owing to their intrinsic biocompatibility, stability, and ability to package diverse molecular cargoes, several research lines are exploring EV engineering strategies for the delivery of targeting and/or therapeutic molecules [6]. In this context, controlled RNA delivery can elicit diverse translational and/or post-transcriptional functions in recipient cells [7] or enable the horizontal transfer of protein–RNA complexes as genome editing tools [8]. However, a desired EV-RNA loading outcome remains challenging due to limited understanding of selective regulatory elements, RNA packaging mechanisms, dosage tolerability, and the number and quality of particles containing the transcript of interest.

Recently, sequence complexity, transcript size, exon number, RNA structuredness, and coding potential have been identified as key parameters influencing EV–RNA sorting [9]. Structuredness and coding probability emerged as significant determinants of circRNA enrichment, and circularization correlated with RNA secretion into EVs [9]. Notably, for these RNA species, the primary sequence composition did not show the same correlations reported for other small non-coding RNAs, such as miRNAs, which are characterized by the enrichment of specific RNA-binding protein recognition motifs that facilitate EV sorting [10,11,12,13].

The role of RNA-binding proteins has also recently emerged for coding transcripts, in association with distinct sub-cellular trafficking dynamics [14,15], and for other pathways that promote the glycosylation of cellular RNAs conveyed into secreted EVs [16]. Collectively, these findings highlight the existence of selective RNA packaging mechanisms.

Recently, we reported that recombinant GFP proteins fused to an N-terminal CD81 could be detected in EVs secreted by transiently transfected HEK293T cells [17]. The proportion of GFP-positive EVs correlated with relative intracellular protein levels, suggesting a certain degree of predictability of the fraction of vesicles containing the protein of interest. In particular, the CD81–GFP fusion protein exhibited higher intracellular fluorescence intensity than a longer construct incorporating a HER2-binding moiety (CD81-antiHER2-GFP or antiHER2), which was associated with an approximately fourfold lower representation of the protein in EVs [17]. Here, we expanded the CD81-guided constructs to test the hypothesis that recombinant mRNA loading into EVs could mirror the intracellular abundance, as observed for the corresponding protein cargo.

## 2. Results

### 2.1. A 126-nt Deletion of CD81 3′-End (CD81Δ) Reduces Fusion Protein Expression

**Figure 1:**
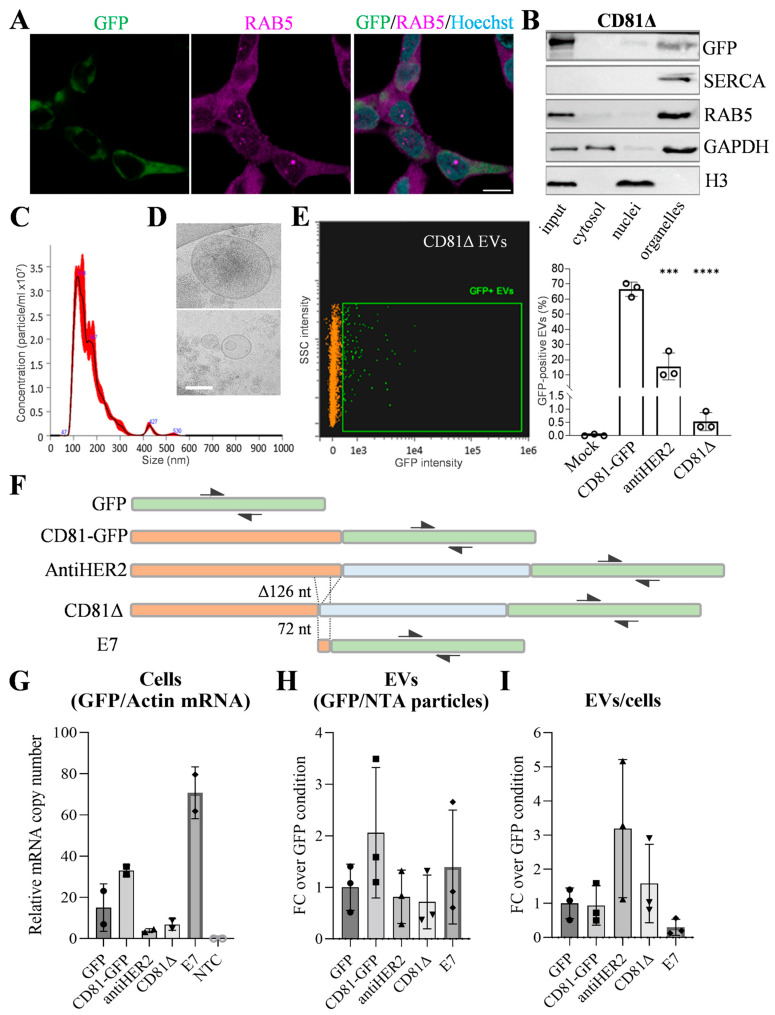
(**A**) Confocal microscopy acquisition of HEK293T cells 48 h after transfection with the CD81Δ construct. Recombinant CD81Δ (CD81Δ-antiHER2-GFP) is visible in green, endogenous RAB5 in magenta (Alexa Fluor568, Thermo Fisher Scientific, Italy), and nuclei in cyan (Hoechst33342, Thermo Fisher Scientific, Italy). Scale bar is 10 μm. (**B**) Immunoblot analysis of sub-cellular fractions obtained by the cell fractionation protocol described in [17]. Fraction quality was confirmed using primary antibodies recognizing GAPDH (cytosol), histone H3 (nuclei), SERCA2 (endoplasmic reticulum), and RAB5 (early endosomes). CD81Δ was detected at the expected molecular weight of ~72 kDa. (**C**) Representative NTA profile of CD81Δ EVs measured by Nanosight N300 [17]. The black line indicates the mean of three independent measurements, with the standard error (SE) shown in red as in the original exported graph. (**D**) Representative cryo-EM images of CD81Δ EVs. Scale bar is 100 nm. (**E**) Imaging flow cytometry (Cytek Biosciences, The Netherlands) analysis of GFP-positive EVs (green dots), performed as previously described [17]. GFP fluorescence (Ch02, 488 nm laser) was detected among particles gated on side scatter (Ch06) and positive to Cell Mask Deep Red (Thermo Fisher Scientific, Italy; Ch11, 635 nm laser, orange dots). Non-fluorescent 1-μm Speed Beads (Amnis, Cytek Biosciences, The Netherlands) were included as acquisition controls. The graph shows quantification of double-positive particles. Data represent the mean ± error from three independent experiments. Statistical analyses were performed to compare with CD81-GFP and antiHER2 EVs already shown in [17]. *** *p* < 0.001; **** *p* < 0.0001. (**F**) Graphical abstract of sequence construct features and lengths used for RNA testing. For all the conditions, transcripts were amplified/detected by ddPCR (Bio-Rad Laboratories, Italy) using the same primer pairs (black arrows). CD81 sequence is depicted in orange; GFP sequence is depicted in light green; Trastuzumab light chains are depicted in light blue. (**G**) GFP mRNA copy number in cells normalized to β-actin mRNA copy number detected in parallel ddPCR reactions. RNA from non-transfected cells (NTC) has been included as a negative biological control. (**H**) GFP mRNA copy number normalized to EV counts. Data are shown as fold change (FC) with respect to the GFP condition. (**I**) Ratio of data reported in H with the average of data in G (EVs/Cells) to highlight the efficiency of GFP transcript loading into EVs relative to intracellular abundance. Data are shown as fold change (FC) with respect to the GFP condition.

To address the relationship between construct length and protein cargo, we derived a new plasmid from the antiHER2 construct. Specifically, the last 126 nucleotides of the CD81 coding sequence were deleted to obtain a partially truncated CD81-antiHER2-GFP construct (CD81Δ). Following the workflow we previously described for the other ectopic proteins [17], we observed that the CD81Δ fusion protein resembled the sub-cellular distribution of the other ectopic proteins, such as accumulation in the same cytoplasmic regions previously described in [17] as partly overlapping with endogenous CD81 or RAB5 proteins (Figure 1A), as well as detection in organelle-enriched fractions after differential sedimentation (Figure 1B). Analogously to the previously characterized CD81-GFP and antiHER2 proteins, these data suggest that the ectopic CD81Δ protein also participates in vesicular trafficking dynamics. However, the fluorescence intensity of the CD81Δ protein was markedly reduced compared to both CD81-GFP and the antiHER2 proteins [17], making the CD81Δ construct an interesting tool to test how reduced intracellular expression can affect ectopic protein enrichment in released EVs.

### 2.2. The Fraction of CD81Δ-Positive EVs Mirrors Intracellular Protein Levels

Nanoparticle tracking analysis (NTA) of particles recovered from serum-free media of HEK293T cells expressing the CD81Δ protein revealed size distribution and concentrations comparable to those observed for the other control conditions (Figure 1C). Consistently, cryo-electron microscopy (Cryo-EM) showed no detectable morphological differences in these EV preparations (Figure 1D). Then, using the same acquisition templates and gating strategy previously described (Cell Mask Deep Red and GFP) [17], we performed imaging flow cytometry of CD81Δ-derived EVs. The percentage of GFP-positive vesicles was significantly reduced compared with the reference GFP proteins already reported in [17] (Figure 1E), further supporting intracellular protein expression levels as a predictive parameter for the relative abundance of GFP-positive EV fractions.

### 2.3. Saturable Distribution of Recombinant Transcripts

To explore whether corresponding recombinant transcripts exhibit the same EV loading according to the coding probability hierarchy of CD81-GFP, antiHER2, and CD81Δ constructs, we isolated cellular and EV-RNA from HEK293T cells transfected with five plasmids, including the GFP alone and E7 encoding vectors. The E7 construct retained 72 nucleotides (58% GC) of the CD81 coding sequence previously deleted in CD81Δ and generated a slightly longer transcript compared with GFP alone. Transcript levels were quantified on cDNA samples by digital droplet PCR (ddPCR) using EvaGreen chemistry and identical GFP primer pairs, as illustrated in Figure 1F. Intracellular GFP mRNA copy number, normalized to actin mRNA levels, was determined in all the samples (except in non-transfected control cells). Under these conditions, the E7 construct (producing detectable protein by immunoblotting in cellular lysates only) exhibited the highest intracellular abundance, followed by CD81-GFP (Figure 1G). In contrast to protein sorting, ddPCR assays on EV-derived RNA revealed no significant differences in recombinant mRNA abundance after normalization to particle numbers measured by NTA (Figure 1H). The EVs/cells RNA ratio indicated a positive trend (>1) for antiHER2 and a negative trend (<1) for E7 mRNAs (Figure 1I), suggesting effects beyond the coding probability if a certain RNA threshold is reached. Moreover, in conditions where the protein cargo mirrors intracellular expression, the variability observed in EV-RNA is modest compared to intracellular transcript levels, limiting our predictive capacity.

## 3. Discussion

We previously designed constructs combining the human full-length CD81 protein with a turbo GFP reporter (CD81-GFP), including or excluding Trastuzumab light chains 1 and 2 (antiHER2), generating proteins of different lengths (45 and 75 kDa, respectively). We found that both fusion proteins are secreted into EVs in proportion to their expression levels in HEK293T cells [17]. Speculating that intracellular expression levels are fundamental for predicting recombinant protein and RNA secretion into EVs, we here generated a shorter CD81Δ construct that yields a fusion protein with lower expression in cells than CD81-GFP or antiHER2, and another construct (E7) that generates a longer transcript than GFP alone. The distribution analysis of cellular and vesicular counterparts suggested that distinct mechanisms sort CD81-guided cargoes, more passive for proteins and more active for RNAs. While the intracellular levels of the fusion proteins positively correlated with the fraction of vesicles carrying the ectopic protein, the corresponding RNA cargo showed unpredictable distribution based on intracellular transcript levels and length. Therefore, EV-RNA sorting mechanisms may depend on the cumulative effects of RNA structure, sub-cellular dynamics, post-transcriptional modifications, and RNA-binding protein interactions, potentially linked to vesicle biogenesis. The main limitations of this study include the lack of evidence for specific substructures within the recombinant transcripts, the RBP manipulation affecting the EV-RNA distribution, and the EV recovery after changing cell culture conditions. However, despite a clear intracellular “availability” of some transcripts, the packaging probability appears saturable, supporting a model in which the concomitant activity of trans-acting factors could dictate the discrete number and quality of vesicles secreted. Therefore, the quantitative and functional characterization of these interaction nodes could be essential to maximize EV-RNA loading strategies.

## 4. Materials and Methods

Cell culture, immunofluorescence, cell fractionation, immunoblotting, EV isolation by differential ultracentrifugation, nanoparticle tracking analyses (NTAs), Cryo-EM, and imaging flow cytometry were performed as already described in [17]. The “organelle” fraction indicated in Figure 1B derives from the cell fractionation experiments already described in [17]. Briefly, the lysis buffer (50 mM HEPES pH 8, 10 mM NaCl, 10 mM MgCl2, 1 mM DTT, 10% glycerol, 1X protease inhibitor cocktail) was supplemented with 25 µg/mL Digitonin (buffer A), 1% Igepal (buffer B) or 1% Triton X-100 and 1% Sodium deoxycholate (buffer C) to perform a sequential incubation and centrifugation protocol at 4 °C. Input samples corresponded to 2% of the whole cell lysate. The first supernatant (cytosolic fraction) was collected after incubation of cells with buffer A, then centrifuged at 2000 rcf for 10 min. The obtained pellet was resuspended and incubated in ice-cold buffer B and then centrifuged at 7000 rcf for 10 min. The resulting supernatant corresponded to the organelle-enriched fraction, while the pellet was resuspended in ice-cold buffer C with the addition of benzonase (Novagen) and incubated on a rotary shaker for 30 min at 4 °C. Next, samples were sonicated for 45 s at 35 Amplitude (three cycles of 10 s on and 5 s off) and centrifuged at 7800 rcf for 10 min to collect the nuclear fraction. Each fraction was loaded on 13% polyacrylamide gel for SDS-PAGE. To be inclusive of both small and large EV populations, we recovered EVs using a standard differential ultracentrifugation procedure. Specifically, we collected the serum-free media after 24 h of incubation with cells and centrifuged at 2800 rcf for 10 min to remove large cell debris and large apoptotic bodies. Subsequently, we subjected the resulting supernatant to centrifugation at 100,000 rcf for 1 h to sediment particles that we finally resuspended in PBS 1X. NTAs were performed as standard acquisitions in the Malvern Nanosight NS300 instrument (Alfatest, Italy) with a 532 nm laser, including three videos of 60 s each, and data were automatically combined in a bidimensional graph using the NanoSight NS300 software NTA 3.4.

### 4.1. Plasmid Cloning

CD81Δ plasmid (CD81Δ-antiHER2-GFP, 7775 bp) was obtained from the antiHER2 construct (CD81-antiHER2-GFP, 7901 bp). Briefly, the cloning strategy comprised antiHER2 vector double digestion with ClaI and MluI enzymes, 5′-overhangs filling-in by DNA polymerase Klenow fragment prior to ligation reaction (all reagents from Thermo Fisher Scientific, Italy).

The E7 plasmid was generated from the CD81-GFP construct. The E7 sequence, corresponding to exon 7 of CD81, was amplified using high-fidelity PCR with the following primers: 5′-GTTATAGCGATCGCCGAGGACTG and 5′-GTAGTGACGCGTGCCGATGAGGTA.

Fragments resulting from digestion with the restriction enzymes MluI and SfaAI were then ligated by T4 DNA ligase (all reagents were from Thermo Fisher Scientific, Italy).

### 4.2. RNA Isolation and ddPCR

Cellular and EV-RNA were extracted using TRIzol™ Reagent (Ambion, Life Technologies, Milan, Italy) in combination with the Single Cell RNA Isolation Kit (Norgen Biotek, Cat. No. 51800, Roma, Italy). RNA was eluted in 30 µL of nuclease-free water for cellular samples and 20 µL of nuclease-free water for EV samples. RNA quantification was performed using a NanoDrop for cellular RNA and a Fragment Analyzer (Agilent Technologies, Italy) for EV-RNA. cDNA synthesis was carried out using the RevertAid First Strand cDNA Synthesis Kit (Thermo Fisher Scientific, Monza, Italy) with 1 µg of cellular RNA and 11 µL of EV-RNA as input.

Droplet digital PCR (ddPCR) was performed using EvaGreen chemistry (QX200™ ddPCR™ EvaGreen Supermix, Bio-Rad Laboratories, Milan, Italy). GFP and β-actin transcripts were respectively amplified with the following primers:

GFP 5′-TGACCTTCAGCCCCTACCT and GFP 5′-CCGTCCTCGTACTTCTCGAT, β-actin 5′-CTGGAACGGTGAAGGTGACA and β-actin 5′-AGGGACTTCCTGTAACAACGCA. For each reaction, cellular cDNA was diluted 1:200, whereas EV-RNA-derived cDNA was diluted 1:80. Droplets were generated using the QX200 AutoDG Droplet Digital PCR System (Bio-Rad). PCR was performed on the T100 Thermal Cycler (Bio-Rad), and droplets were detected using the QX200™ Droplet Reader (Bio-Rad Laboratories, Italy). To assess the relative distribution of specific transcripts into secreted EVs, we included the relative amount of particles acquired by NTA, the same input RNA amount for cDNA synthesis, and the relative expression level of the target at the intracellular level.

## Data Availability

Data used to generate Figure 1 are available from the corresponding author upon reasonable request.
